# Chronic Lactation Insufficiency Is a Public Health Issue: Commentary on “We Need Patient-Centered Research in Breastfeeding Medicine” by Stuebe. Breastfeed Med 2021;16:349–350.

**DOI:** 10.1089/bfm.2021.0202

**Published:** 2021-12-07

**Authors:** Helen Shere, Laurel Weijer, Harriet Dashnow, L. Elizabeth Moreno, Susanna Foxworthy Scott, Helen Baker

**Affiliations:** ^1^ Low Milk Supply Foundation Board, Gainesville, Florida, USA.; ^2^ Department of Medical Student Education, Indiana University School of Medicine, Indiana University, Indianapolis, Indiana, USA.; ^3^ Department of Epidemiology, University of Florida, Gainesville, Florida, USA.; ^4^ Department of Human Genetics, University of Utah, Salt Lake City, Utah, USA.; ^5^ Department of Family and Preventive Medicine, University of Utah, Salt Lake City, Utah, USA.; ^6^ Department of Communication Studies, Indiana University-Purdue University Indianapolis, Indianapolis, Indiana, USA.; ^7^ Lillian Carter Center for Global Health and Social Responsibility, Nell Hodgson Woodruff School of Nursing, Emory University, Atlanta, Georgia, USA.

The President's Corner article by Dr. Alison Stuebe entitled “We Need Patient-Centered Research in Breastfeeding Medicine” shines a light on the need for increased health care provider awareness, education, and empathy in clinical cases wherein exclusive breastfeeding is not able to be achieved.^1^ For lactating parents experiencing chronic lactation insufficiency—here defined as the production of less milk than is required to exclusively feed an infant for the duration of the breastfeeding relationship, despite following best practices—issues around health care provider awareness, along with a lack of evidence-based solutions, often lead only to increased frustration during an already fraught time for parents' physical and mental well-being.

We are members of the Low Milk Supply Research Association, a consortium of researchers, scientists, lactation, and health care professionals who have themselves experienced chronic lactation insufficiency. Several of the authors are also founding members of the newly launched Low Milk Supply Foundation, a nonprofit organization dedicated to addressing the gap in research and knowledge about, and support for, chronic lactation insufficiency. In the article, Dr. Steube writes that “…lactation, like every other physiologic process, can malfunction. To repair that malfunction, we need the tools to diagnose the underlying pathophysiology and an evidence base for treatment.”^1^ We commend Dr. Stuebe for bringing chronic lactation insufficiency into the light. Yet, we lament that this widespread issue has remained largely ignored in the research literature. The time has come for chronic lactation insufficiency to be recognized and researched as a public health problem.

It is estimated that 5–15% of breastfeeding parents experience chronic lactation insufficiency.^2^ Given the ∼3.6 million live births in the United States in 2020, we can extrapolate that this issue affected anywhere from 180,000 to >500,000 breastfeeding dyads in the past year alone.^3^ These parents are not able to feed their children the biologically optimal source of nutrition for infants, nor are they able to follow breastfeeding recommendations from major health organizations such as the World Health Organization, Centers for Disease Control and Prevention, and the American Academy of Pediatrics. The children of parents affected by chronic lactation insufficiency are also possibly missing the immune system benefits afforded by breastfeeding—a lack acutely felt during the COVID-19 pandemic. Finally, parents who plan to breastfeed and find themselves unable to do so often feel betrayed by their bodies. They may feel judged by family, peers, and health care providers, and hopeless about their lack of choice in how they feed their infants. This mélange of emotions may increase the risk for postpartum mood disorders, another growing public health concern.

A greater understanding of the causes of and risk factors for chronic lactation insufficiency may lead to improved long-term health outcomes for women. Chronic lactation insufficiency has been associated with a number of conditions, including thyroid disorders, polycystic ovary syndrome (PCOS), insulin resistance, hormonal imbalances, micronutrient deficiencies, and insufficient glandular tissue (IGT).^4–6^ Given that 1 in 10 women of childbearing age is affected by PCOS, 1 in 8 women experiences thyroid dysfunction, and rates of insulin resistance, prediabetes, and type-2 diabetes are on the rise, it is possible that chronic lactation insufficiency may be the first sign of one or multiple of these conditions. Rather than brushing patients off when they present with chronic lactation insufficiency, health care providers should use this as an opportunity to take a deeper look at their patients' long-term health in a supportive way.

There are protocols and articles in breastfeeding and pediatric journals related to the evaluation of low milk supply related to appropriate weight gain in infants.^7,8^ But as of this writing, official screening and diagnostic criteria for chronic lactation insufficiency do not exist, nor does it exist for IGT or breast hypoplasia. To further complicate diagnosis, IGT and breast hypoplasia are often conflated in the literature, and although IGT is a known cause of chronic lactation insufficiency, breast hypoplasia has not been identified as a cause. Some parents with hypoplastic breast appearance can produce a full supply, whereas parents with insufficient supply and capacity can have a typical breast appearance.^9^ This lack of consistent diagnostic and screening criteria, in addition to inadequate education for health care providers related to this area, often leaves individuals navigating this challenge alone, without the support or guidance of their health care providers.

Finally, there is a general gap in understanding regarding the pathophysiology of chronic lactation insufficiency. A PubMed search for the keywords “low milk supply” and “lactation insufficiency” returns 1,795 and 1,815 results, respectively. In contrast, a search for the term “erectile dysfunction” returns 26,582 results. Erectile dysfunction and chronic lactation insufficiency are both serious conditions that can significantly affect patients' quality of life (and in the case of chronic lactation insufficiency, multiple lives) and can indicate a higher risk for or co-occurrence of serious health conditions. Why is it that one condition receives funding and space for research, whereas the other's very existence goes unrecognized?

According to the World Alliance for Breastfeeding Action website, this year's theme for World Breastfeeding Week is “Protect Breastfeeding: A Shared Responsibility.” If we are to truly share the responsibility for helping every infant access optimal nutrition, the time for quality research into the pathophysiology of chronic lactation insufficiency is now. In a 2012 blog post, Dr. Steube wrote, “It's time to stop bickering about whether this mom tried as hard as that mom to breastfeed. We have too much work to do.”^10^ We must establish strategies to identify parents at risk prenatally, as well as diagnostic criteria and standard screening guidelines for health care providers working with postpartum patients with chronic lactation insufficiency. Just as urgent is the need for health care provider education about chronic lactation insufficiency, so that instead of dismissal, affected parents can be met with empathy and guided to skilled support that is dyad centered and evidence based.
